# Anti-inflammatory potential of the CISACN adduct in LPS-induced murine models

**DOI:** 10.1007/s10787-026-02280-9

**Published:** 2026-06-02

**Authors:** Louise M. de Lima, Francisco A. A. F. Gadelha, Laércia K. D. Paiva Ferreira, Cosmo I. D. Vieira, João B. de Oliveira, Larissa A. M. Paiva Ferreira, Matheus Victor de Souza Laurentino, Bruno Vinícius da Silva Moura, Teresa Carolliny M. L. Rodrigues, Tayná R. Olegário, Luciana Scotti, Marcus T. Scotti, Claudio G. Lima-Junior, Naiara N. Dejani, Marcia R. Piuvezam

**Affiliations:** 1 Laboratório de Imunofarmacologia, Programa de Pós-Graduação em Produtos Naturais e Sintéticos Bioativos, Centro de Ciências da Saúde, Universidade Federal da Paraíba, João Pessoa, PB Brazil; 2 Laboratório de Quimioinformática, Programa de Pós-Graduação em Produtos Naturais e Sintéticos Bioativos, Centro de Ciências da Saúde, Universidade Federal da Paraíba, João Pessoa, PB Brazil; 3 Laboratório de Síntese Orgânica Medicinal da Paraíba, Departamento de Química, Centro de Ciências Exatas e da Natureza, Universidade Federal da Paraíba, João Pessoa, PB Brazil; 4 Laboratório de Imunfarmacologia, Instituto de Pesquisa em Fármacos e Medicamentos, Universidade Federal da Paraíba, João Pessoa, PB Brazil; 5 Complexo de Ciências Médicas e de Enfermagem, Universidade Federal de Alagoas, Arapiraca, AL Brazil

**Keywords:** Anti-inflammatory activity, Acute lung injury, Lipopolysaccharide, Cytokines, molecular targets

## Abstract

Acute lung injury (ALI) is characterized by a severe inflammatory response that triggers pulmonary edema, reduced lung compliance, and hypoxemic respiratory failure. To date, there is no effective standard pharmacotherapy for this condition. Recent studies have demonstrated that the Morita-Baylis–Hillman adduct (CISACN) exhibits anti-inflammatory activities in models of asthma and allergic rhinitis, alongside antitumor and antimalarial properties. It also presents low toxicity, low cytotoxicity, and good oral availability. Therefore, this study aimed to investigate the anti-inflammatory activity of the CISACN adduct in a lipopolysaccharide (LPS)-induced ALI model. BALB/c mice were challenged by nasal instillation with LPS and orally treated with CISACN (6.25, 12.5, or 25 mg/kg). Twenty-four hours later, bronchoalveolar lavage fluid (BALF), serum, and lung tissue were collected. Treatment with CISACN, primarily at a dose of 12.5 mg/kg, significantly reduced lung inflammation, as evidenced by decreased cell migration, levels of TNF-α, IL-1β, and IL-6, as well as reduced protein exudate in BALF. Additionally, it decreased serum TNF-α levels and attenuated histopathological alterations in the lung, such as edema, inflammatory cell infiltration, and hemorrhage. The anti-inflammatory effect of CISACN was related to its high energy and affinity with TLR4/p38 MAPK molecules, as evidenced by an in silico approach and confirmed by in vitro analysis, which showed inhibition of p38 MAPK activity. Therefore, this study highlights the anti-inflammatory potential of CISACN by inhibiting a key inflammatory pathway, identifying it as a promising agent for treating inflammatory processes.

**Graphical abstract:**

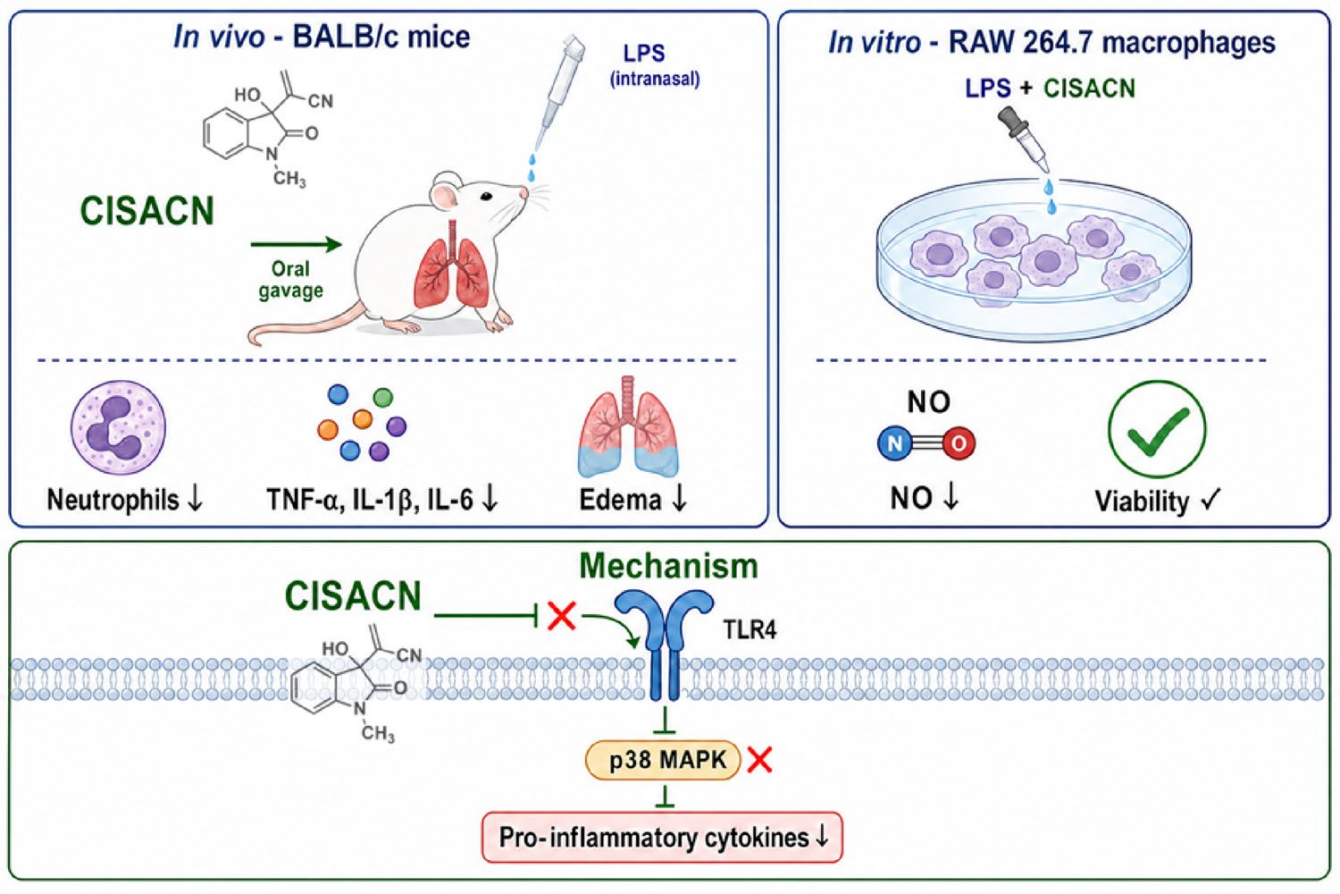

**Supplementary Information:**

The online version contains supplementary material available at 10.1007/s10787-026-02280-9.

## Introduction

Acute lung injury (ALI) and its most severe form, acute respiratory distress syndrome (ARDS), are inflammatory diseases characterized by dense neutrophil infiltration, diffuse bilateral pulmonary edema with decreased lung compliance, alveolar damage, and deposition of the hyaline membrane in the bronchoalveolar lumen, resulting in hypoxic respiratory failure (Zheng et al. 2024).

Due to the lack of effective pharmacological protocol, the mortality in ARDS is around 33% worldwide making it responsible for more than 10% of all intensive care unit admissions, and 4% of all hospital admissions resulting in a significant demand for healthcare resources (Chen et al. 2024). In 2020, ARDS has gained greater prominence due to its association with the COVID-19 pandemic state, as the more severe form of COVID-19 leads to life-threatening ARDS (Lim et al. 2023). However, there are other ARDS origins categorized into two large groups: direct pulmonary causes, which occur when the damage reaches the lungs through the airways or chest trauma; and indirect pulmonary causes, when the agent causing the damage, reaches the lungs through the bloodstream, as seen in sepsis (Bos and Ware 2022).

Thereby, to study potential drug trials to treat ALI/ARDS, several experimental models have been developed (Dhege et al. 2024), and one of them uses lipopolysaccharide (LPS) as the inductor of ALI in mice (Tian et al. 2023). The endotoxin activates the TLR4 (toll-like receptor 4)/CD14/MD2 (myeloid differentiation factor 2) complex and it triggers an intracellular signaling cascade mediated by MyD88 (myeloid differentiation primary response protein) and p38, ERK1/2 and JNK MAPKs (mitogen-activated protein kinases), NF-κB (nuclear factor kappa-B) activation, which is responsible for the expression of several genes involved in the inflammatory process, including the pro-inflammatory cytokines (Mukherjee and Bayry 2024).

In physiological processes, p38 MAPKs are involved in signaling pathways such as cell growth, DNA repair, and cell differentiation. However, the main activity of p38 is in the inflammatory process. After its double phosphorylation, p38 can activate several proteins and cellular transcription factors, including NF-κB, which, in acute lung injury, leads to the production and release of cytokines like IL-1β, INF-α, and IL-6, enzymes like iNOS (inducible nitric oxide synthase) and SOD (superoxide dismutase), and adhesion molecules such as ICAM (intercellular adhesion molecule) and VCAM (vascular cell adhesion molecule), resulting in intense lung damage (Bedard-Matteau et al. 2024).

**Fig. 1:**
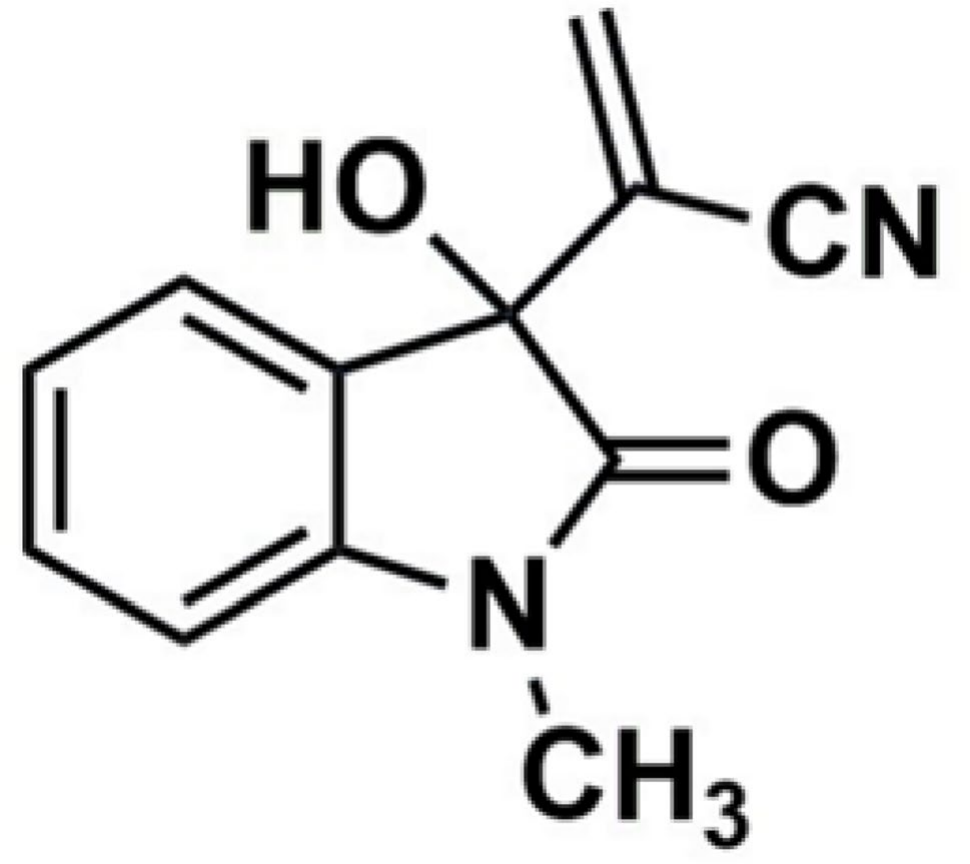
The chemical structure of the CISACN [2-(3-hydroxy-1-methyl-2-oxoindolin-3-yl) acrylonitrile]

In this context, medicinal chemistry with organic synthesis has demonstrated a significant impact on drug discovery and development to treat life-threatening conditions (Krishnamoorthy et al. 2023; Pallavi et al. 2023). Therefore, the Morita-Baylis–Hillman reaction (MBHR), a highly advantageous methodology, synthesizes products called Morita-Baylis–Hillman adducts (MBHA) with remarkable synthetic versatility and a broad spectrum of biological activities (Ferreira et al. 2023). The 2-(3-hydroxy-1-methyl-2-oxoindolin-3-yl) acrylonitrile (CISACN, Fig. 1) is an example of MBHA that presents biological activities, including anti-inflammatory activity in a model of asthma and allergic rhinitis, antitumor effect against the human leukemia cell line HL-60 and the lung cancer cell line NCI-H292, and antimalarial activity, with reduction of *Plasmodium falciparum* (W2) in human erythrocytes. The adduct also presented low acute toxicity and cytotoxicity with good theoretical oral availability (Lima et al. 2016; Brito et al. 2020; Ferreira et al. 2024).

Therefore, the therapeutic potential of CISACN led us to investigate its anti-inflammatory effect in the lipopolysaccharide-induced ALI model by analyzing whether it attenuates the lung inflammatory process.

## Material and methods

### Morita-Baylis–Hillman adduct

The Morita-Baylis–Hillman adduct (CISACN-[2-(3-hydroxy-1-methyl-2-oxoindolin-3-yl) acrylonitrile]) was synthesized according to the previously described methodology (Lima et al. 2016). Two synthetic steps were performed, one involved the isatin methylation through an SN_2_-type reaction using K_2_CO_3_ as the base, and methyl iodide as the alkylating agent, with a yield of 98% of the product. Subsequently, the intermediate *N*-methyl-isatin was subjected to the Morita-Baylis–Hillman reaction using acrylonitrile as Michael acceptor and DABCO as a catalyst, forming CISACN in 95% yield after 90 min of reaction.

### Animals

Isogenic male BALB/c mice (6 to 8 weeks old) weighing between 20 and 25 g were provided by the Keizo Asami Laboratory of Immunopathology (LIKA) of the Federal University of Pernambuco, Recife, PE, Brazil. Animal model was approved by the Ethics Committee on Animal Use under certificate number 7316150420. The animals were kept in polypropylene cages at a temperature of 21 ± 2 °C, in light and dark cycles of 12 h with free access to water and a controlled diet based on pellet feed throughout the experimentation period. The animals were euthanized by intramuscular administration of an anesthetic solution containing 29 mg/mL ketamine and 1.91 mg/mL xylazine in saline solution.

### Lipopolysaccharide (LPS)—induced acute lung injury (ALI)

The experimental protocol for ALI was developed and adapted from the previously described methodology (Bernardo et al. 2022). The animals (*n* = 6 per group) were intramuscularly anesthetized and LPS-challenged, by nasal instillation, with 40 µL of 2.5 mg/kg of lipopolysaccharide (LPS—*Escherichia coli* O111: B4; Sigma Aldrich). One hour later, LPS-challenged animals were orally treated (gavage) with CISACN at 6.25, 12.5, or 25 mg/kg. The basal group was treated with vehicle. After twenty-four hours, the animals were euthanized, and the biological material was collected for analysis.

### Cell quantification

The inflammatory cell migration to the lung cavity was measured in the bronchoalveolar lavage fluid (BALF). The BALF was harvested with 5 mL of cold PBS injected into the animal lung through the trachea, and the fluid was transferred to microtubes and stored at 4 °C. Total cell count was performed in a hemocytometer chamber (Turk’s solution 1:10) under the optical microscope (40 X–BX40, OLYMPUS). The cell supernatants were used for subsequent protein and cytokine dosages. The differential cell counting was performed by cytospin centrifugation, and the slides containing the cells were fixed and stained with the panoptic methodology. Each slide was analyzed using the immersion objective under the optical microscope (100 X–BX40, OLYMPUS) (Cavalcanti et al. 2023).

### Total protein assay

The total protein content in the BALF samples was quantified using the colorimetric red pyrogallol method with the commercial SENSIPROT kit (Lab Test, Minas Gerais, MG, Brazil), according to the manufacturer’s specifications. Briefly, samples and a serial dilution of a bovine serum albumin (BSA) standard were incubated with the working solution. The resulting colorimetric reaction was measured in a spectrophotometer at a wavelength of 600 nm. The total protein concentration for each sample was determined by interpolation from the standard curve.

### Wet/dry lung weight ratio

The wet/dry (W/D) lung weight ratio was determined to assess pulmonary edema. Immediately after collection, the lung was isolated, carefully dried of superficial fluid, and weighed to obtain the wet weight. Subsequently, the tissue was incubated in an oven at 60 °C for 72 h until a constant dry weight was achieved. The W/D ratio was then calculated by dividing the initial wet weight by the final dry weight. This method is widely used to quantify tissue water content, with a higher ratio indicating greater edema formation (Ferreira et al. 2022).

### Cytokine quantification

The measurement of IL-1β, TNF-α, and IL-6 cytokines in BALF and peripheral blood was performed using enzyme-linked immunosorbent assay (ELISA) commercial kits, following the manufacturer’s specifications (eBioscience, San Diego, California, USA). Briefly, samples and standards were added in duplicate to pre-coated plates. After incubation and washing steps, a biotin-conjugated detection antibody was added, followed by streptavidin-HRP. The reaction was developed with TMB substrate, stopped with stop solution, and the absorbance was read at 450 nm. Cytokine concentrations were determined by interpolation from the respective standard curves.

### The lung histological analysis

To evaluate the histological aspect of the lungs, they were collected 24 h after the LPS challenge and fixed in buffered formalin, hydrated, and dehydrated in ethyl alcohol, xylol, and liquid paraffin (histological paraffin—ERVIEGAS, São Paulo, SP, Brazil). The paraffin block was cut into sections of 5 μm in a microtome (SP Labor 300). Tissue sections adhered to the slides were stained with Hematoxylin & Eosin (H&E). The morphological tissue analysis was performed using a histological score as follows: 0—normal (no changes); 1—mild (changes in less than 25% of the microscopic field); 2—moderate (changes between 25%—50% of the microscopic field); 3—severe (changes between 50%—75% of the microscopic field); or 4—extremely severe (changes in more than 75% of the microscopic field). In each slide, 10 fields were analyzed, and the final score was calculated using the average of these fields. The histological changes were cell infiltration, edema formation, and hemorrhage (Paiva Ferreira et al. 2021).

### Molecular docking

The molecular structures of CISACN and dexamethasone were drawn in Marvin Sketch 21.13 and optimized in HyperChem 8.0.6 software (RMS 0.1 kcal/mol/Å). Standard settings were used, applying the force field method of molecular mechanics MM+ and semi-empirical quantum method AM1 (Austin Model 1) (Scotti et al. 2009; Rodrigues et al. 2021; Abbasi et al. 2022). The crystal structures of the proteins were obtained from the RCSB Protein Data Bank (PDB) (http://www.rcsb.org/pdb/home/home.do) with resolution up to 3 Å, under codes for p38 MAPK—1A9U, ERK 2 MAPK—4QPA, NF-ĸB p65 portion—1K3Z, and TLR4—2Z64. The docking of the molecules with the targets was performed in the Molegro Virtual Docker (MVD) software, the parameters used to configure the software were: for the binding energy score values, MolDock Score and Rerank Score followed by the arithmetic mean of two points with the cutoff value of > − 8 kcal/mol; for the GRID, the resolution of 0.3 Å and a spherical radius of 15 Å; for the ligand evaluation, the internal ES, internal HBond, Sp2-Sp2 twists; and for the MolDock Simplex Evolution (MolDock SE) algorithm, run with 10 and maximum of 1,500 interactions (Thomsen and Christensen 2006). To validate the technique, redocking was performed and values for root mean square deviation (RMSD—Root Mean Square Deviation) < 2 Å were analyzed to determine the average overlapping distance of the protein–ligand atoms (Schlosser and Rarey 2009). Other evaluated parameters were the anchorage energy and the types of interactions with the target-binding site. Finally, the 2D and 3D images of the ligand interactions with the targets were viewed using the Biovia Discovery Studio Visualizer 19.1.0.18287 program (Vijayakumar et al. 2020).

#### RAW 264.7 cell line

The RAW 264.7 cell line is a mouse leukemic monocyte-macrophage. The Raw cells, used in vitro experiments, were obtained from the Rio de Janeiro Cell Bank (BCRJ/UFRJ). The cells were maintained in cell culture medium DMEM containing 10% FBS (fetal bovine serum) and streptomycin/penicillin (1:1000) at 37ºC in an atmosphere with 5% CO_2_, following the American Type Culture Collection guidelines. RAW 264.7 cells (5 × 10^4^/well) were distributed in 96-well plates or (5 × 10^5^/well) 24-well plates and used for pretreatment and posttreatment assays. The CISACN was solubilized in DMSO and then diluted in DMEM culture medium. The concentrations used were based on previous studies as isatin-derived molecules. Pretreatment protocol: CISACN (5, 10, and 20 μM) was added to the cell culture 2 h before LPS-stimulation (1 μg/mL). Post-treatment protocol: cells were LPS-stimulated (1 μg/mL) and, after 2 h, CISACN (5, 10, and 20 μM) was added and incubated for 24 h at 37ºC and in 5% CO_2_. After this time, cell viability was analyzed using the MTT assay or Flow cytometry, and supernatants were collected for nitrite quantification. The nitrite level (NO₂ stable product) was measured in the cell culture supernatant samples by Griess reagent as previously described (Gadelha et al. 2021). The absorbance of the samples was measured in a microplate reader at 540 nm (Biotek, model 800 TS).

#### Flow cytometry to p-p38 MAPK

After CISACN treatments, cells were collected, fixed, and permeabilized using the BD Phosflow™ Fix Buffer I and BD Phosflow™ Perm Buffer III, following the manufacturer’s instructions. Then, cells were treated with anti-CD16/CD32 to block non-specific FC-mediated interactions and then marked with anti-p-P38 MAPK (Thermo Fisher Scientific-Waltham, MA, USA), according to the manufacturer’s instructions. Finally, cells were suspended in PBS and evaluated using a BD FACSCanto II flow cytometer (Becton Dickinson and San Jose, CA) and FACS DIVA software.

#### Statistical analyses

All data are expressed as mean ± standard error of the mean (SEM) as indicated. Statistical analyses were performed using one‑way analysis of variance (ANOVA) followed by Tukey’s post‑hoc test for multiple comparisons, or Student’s t‑test for two‑group comparisons, as appropriate. A *p*‑value < 0.05 was considered statistically significant. All statistical analyses were performed using GraphPad Prism software.

## Results

### Effect of CISACN on inflammatory cells and mediators

**Fig. 2:**
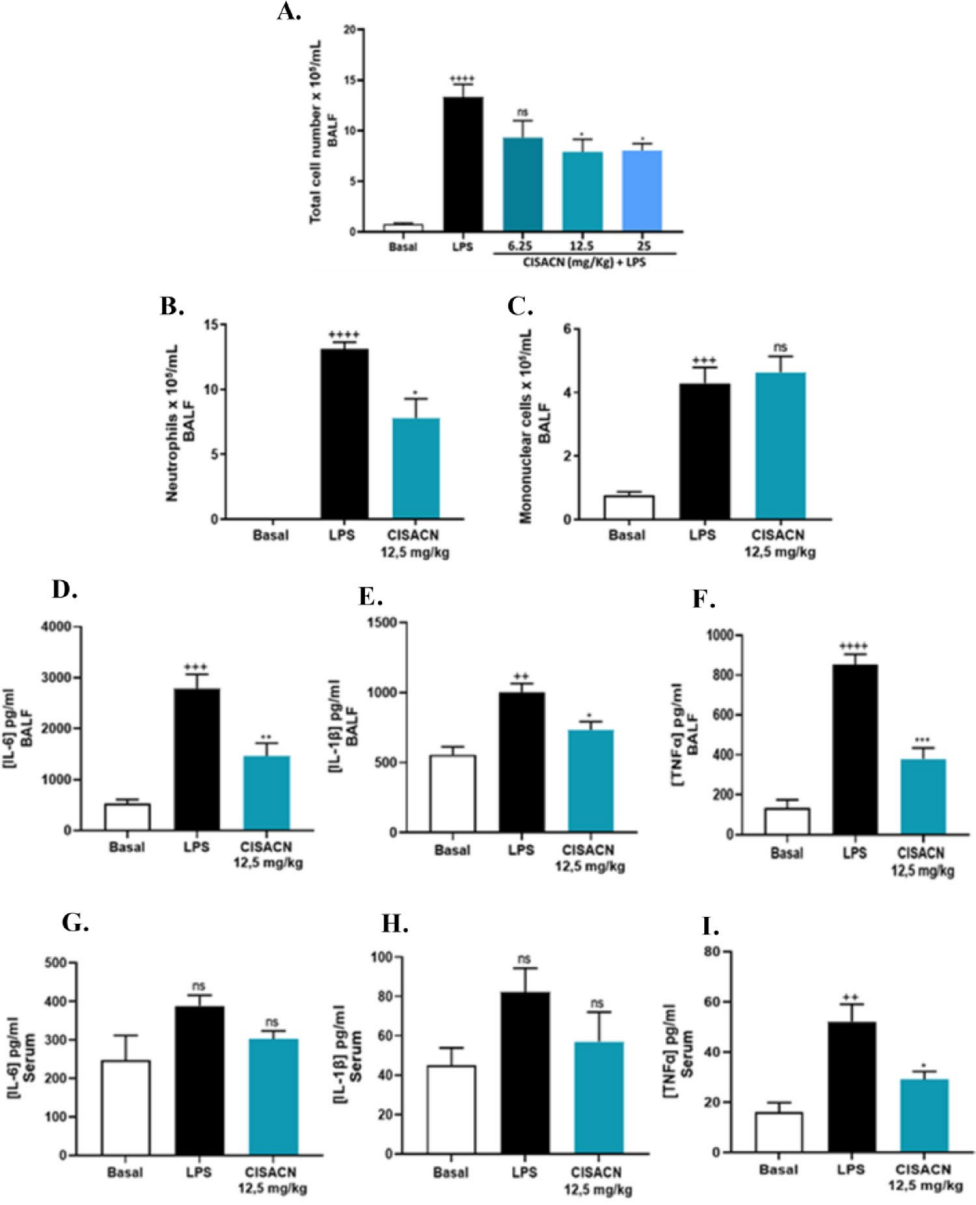
Effect of CISACN on the inflammatory cell migration to the pulmonary cavity, and BALF and serum cytokines levels in LPS-induced acute lung injury. Male BALB/c mice (*n* = 6 per group) were challenged with LPS and orally treated with CISACN. Twenty-four hours later, the bronchoalveolar lavage fluid (BALF) was collected for total and differential inflammatory cell counting and cytokine quantification by ELISA. **A** Total cells, **B** neutrophils, **C** mononuclear cells. BALF: **D** IL-6; **E** IL-1β; **F** TNF-α. Serum: **G** IL-6; **H** IL-1β; **I** TNF-α. The data represents the mean ± S.E.M. The data were analyzed by one-way ANOVA, followed by Tukey’s post-test. + ++*p* < 0.0001, ++++*p* < 0.00001 (compared with Basal); *p < 0.05 (compared with LPS); ns (not significant)

The cell profile in the BALF of the animal groups is shown in Fig. 2. The LPS group showed an increase in total inflammatory cells in the perialveolar space, as expected. CISACN treatment (12.5 mg/kg or 25 mg/kg) significantly attenuated cell migration compared with the LPS group (Fig. 2A). Therefore, 12.5 mg/kg was selected for further study. Regarding the differential inflammatory cell count, the LPS group exhibited an increase in the majoritary cells, which are neutrophils and also in mononuclear cells in the perialveolar space, and CISACN treatment mainly reduced the neutrophil migration independent of mononuclear cells (Fig. 2B, C).

The IL-6, IL-1β, and TNF-α levels in BALF and serum are shown in Fig. 2D–I. The LPS group showed an increase of IL-6, IL-1β, and TNF-α levels in the BALF, and the CISACN (12.5 mg/kg) treatment reduced these cytokine levels. The LPS group also exhibits an increase in TNF-α serum level, regardless of the other cytokines (Fig. 2G–H). The CISACN treatment reduced the TNF-α serum level compared to the LPS group (Fig. 2I).

### Effect of CISACN on lung tissue

**Fig. 3:**
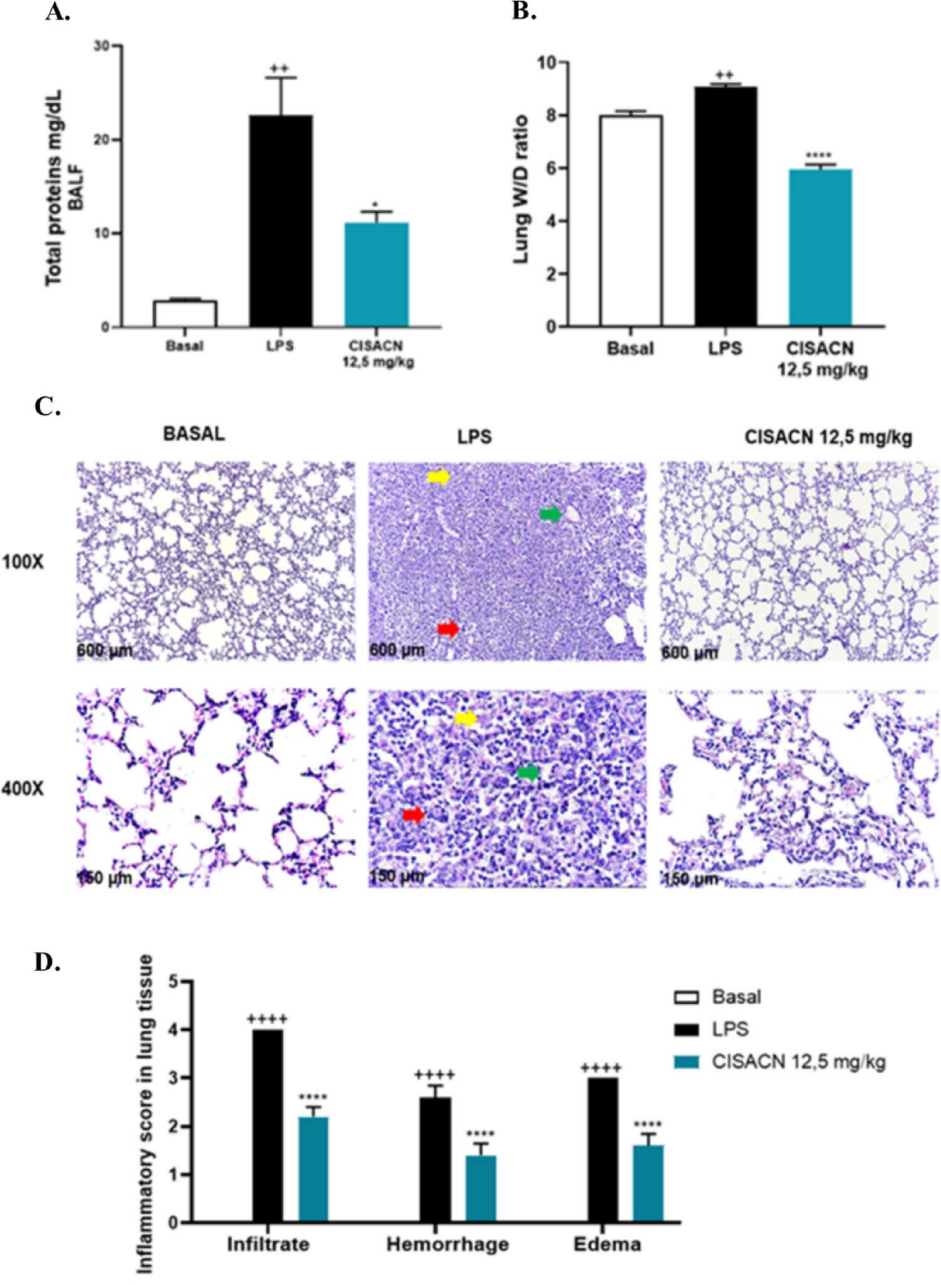
Effect of CISACN on the lung of the LPS-induced acute lung injury model. BALB/c mice (n = 6) were LPS-challenged and orally treated with CISACN. Twenty-four hours later, the bronchoalveolar lavage fluid (BALF) and the lung were collected for total protein quantification and histological analysis, respectively. Total protein content (**A**); the lung weight ratio (W/D-wet/dry) (**B**); lung photomicrographs at magnifications of 100× (600 µm) and 400x (150 µm), red arrows (cellular infiltrate), yellow arrows (hemorrhage) and green arrows (edema) (**C**); and inflammatory score (**D**). The data were analyzed by one-way ANOVA, followed by Tukey’s post-test. +++*p* < 0.0001, ++++*p* < 0.00001 (compared with Basal); *p < 0.05 (compared with LPS); ns (not significant)

The lung damage was visualized by histological analysis, vascular permeability by BALF protein quantification, and W/D ratio by weighing the lung before and after drying. The LPS group showed an increase in the protein content, while the CISACN group (12.5 mg/kg) reduced BALF protein levels (Fig. 3A). In addition, there was an increase in the W/D ratio in the LPS group compared with the basal group, and the treatment with CISACN (12.5 mg/kg) significantly decreased the W/D ratio compared with the LPS group (Fig. 3 B).

The lung histological aspects are demonstrated by photomicrographs (100× and 400×), in Fig. 3C. The basal group showed preserved lung histological characteristic, however, LPS group presented intense inflammatory cellular infiltration (red arrow), alveolar edema with hyaline membranes and thickening of the alveolar wall (green arrow), and clusters of red blood cells (hemorrhage) (yellow arrow). The CISACN treatment (12.5 mg/kg) decreased alveolar inflammation by reducing inflammatory cell migration, edema, and hemorrhage, and a decrease in diffuse alveolar damage. By histological score, the inflammatory parameters were reduced with CISACN treatment (Fig. 3D).

### Effect of CISACN on RAW 264.7 cells

**Fig. 4:**
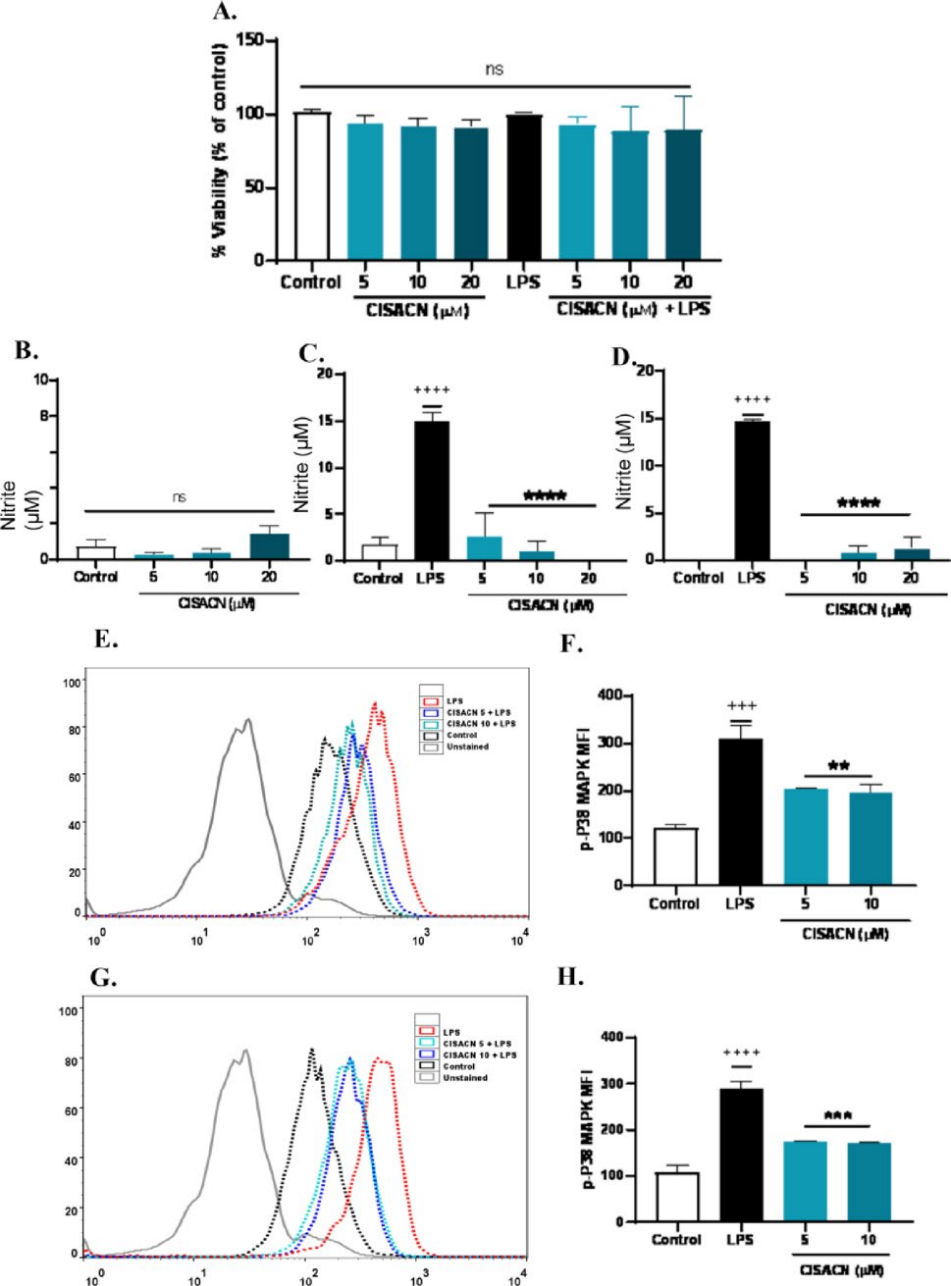
Effect of CISACN on RAW 264.7 cells with or without LPS-stimulus. Unstimulated and LPS-stimulated cells were exposed to different concentrations of CISACN (5, 10, or 20 µM) for 24 h. Cell viability (**A**), Nitrite on unstimulated cells (**B**), CISACN pre-treated (**C**); post-treated (**D**) LPS-stimulation. The Mean Fluorescence Intensity (MFI) was also analyzed: pre-treated (**F**) and post-treated (**H**) LPS-stimulation. The p-P38 MFI histograms: pre-treated (**E**) and post-treated (**G**) LPS-stimulation. Cell viability was assessed by MTT method. The supernatants were used for nitrite production analysis by Griess method. The cells were stained with an anti-p-P38 antibody, and at least 10,000 events were analyzed. Data represents the mean ± S.E.M. and was analyzed by one-way ANOVA followed by Tukey’s or Bonferroni’s post-test. ++*p* < 0.001, +++*p* < 0.0001, ++++*p* < 0.00001 (compared to control group); *p < 0.05, **p < 0.001, ***p < 0.0001 (compared to the LPS group); ns (not significant)

RAW 264.7 cell viability in the presence of CISACN (5, 10, or 20 µM) is shown in Fig. 4. CISACN, either alone or in the presence of LPS, did not alter the cell viability (Fig. 4A). The IC50 was determined from the range of 2.5 to 40 µM in RAW cells, being defined as 22.68 µM. (Data not shown). Also, CISACN did not induce nitrite production in this cell line (Fig. 4B). However, both pre- and post-treatment with CISACN significantly reduced the nitric production previously induced by LPS (Fig. 4C, D).

In addition, LPS-stimulated RAW 264.7 cells exhibited a significant increase in phosphorylated P38 (p-P38) expression compared to non-LPS-stimulated cells (Fig. 4E, F). Both pre- and post-treatment with CISACN (5 or 10 μM) significantly reduced the mean fluorescence intensity (MFI) for p-P38 compared to the LPS-stimulated cells (Fig. 4E–H). However, neither pre- nor post-treatment altered the percentage of p-P38 positive cells (supplementary material).

## Discussion

Acute lung injury (ALI) is a severe and potentially fatal condition that affects lung complacency. The inflammatory response caused by several injuries plays a crucial role in the progression of the disease, which is characterized by extensive cellular infiltration, edema, and production of pro-inflammatory cytokines, favoring hypoxic conditions (Zhu et al. 2023). In the search for new therapies that minimize the inflammatory response and lung damage, CISACN, an isatin-derived MBHA, stands out due to the antitumor and antimalarial activities previously described (Lima et al. 2016, Brito et al. 2020, Ferreira, Ferreira et al. 2024).

This study evaluated the anti-inflammatory effect and the presumable mechanisms of action of CISACN in an LPS-induced ALI model. The results demonstrated that CISACN significantly attenuated lung inflammatory condition by inhibiting the inflammatory cell migration to the lung and the production of pro-inflammatory cytokines attenuating alveolar damage. The *in-silico* study demonstrated a high-energy interaction with TLR4 and p38 MAPK, characterizing possible inflammatory pathway modulation (supplementary material). Regarding this information, the in vitro assays showed the adduct inhibited p38MAPK activity.

The ALI characteristic inflammation involves extensive alveolar leukocyte migration, mainly neutrophils, which lead to the release of inflammatory mediators, such as cytokines, proteolytic enzymes, and reactive oxygen species, that, in excess, aggravate the diffuse lung injury (Song et al. 2022; Xu et al. 2022). The CISACN reduced the lung neutrophil migration induced by LPS, maintaining the alveolus integrity. Regarding these data, a study using a combined allergic rhinitis and asthma syndrome (CARAS) experimental model and CISACN as a testing drug demonstrated a decrease in eosinophil migration to the nasal and lung cavities and tissues, attenuated airway hyperactivity by reducing the hyperplasia/hypertrophy of the smooth muscle, and in lung granulocytes, decreased the p-p38MAPK/p65NF-κB activation (Ferreira et al. 2024).

In addition to the intense neutrophil infiltration and the alveolus damage, the epithelial and endothelial damage leads to the breakdown of the alveolar-epithelial-endothelial barrier and, consequently, edema formation and impairment of lung compliance in ALI (Wang et al. 2024a). After the CISACN treatment, the animals with ALI showed protein exudate and pulmonary edema reduction, conferring an anti-edematogenic property of the molecule.

High levels of inflammatory cytokines such as IL-1β, IL-6, and TNF-α in BALF and serum indicate a small probability of animal survival in the ALI model (Kim et al. 2023). We observed that CISACN treatment reduced the level of these cytokines in the BALF. However, at a systemic level, only TNF-α was decreased with the treatment, which may be due to the period chosen for the analysis.

TNF-α is one of the first inflammatory cytokines to be released by LPS-stimulated macrophages and is responsible for the secretion of other inflammatory cytokines/chemokines that recruit polymorphonuclear leukocytes into the damaged tissue and further exacerbate lung injury (Liu et al. 2022). Similarly, IL-1β production by proteasome activation in LPS-stimulated macrophages also stimulates the release of chemotactic cytokines, e.g., IL-6, that act together in a cascade to amplify the inflammatory process (Tu et al. 2023). For example, IL-6 has been considered an important serum marker in the COVID-19 disease causing acute respiratory distress syndrome (ARDS) (Mollazadeh et al. 2024).

Therefore, the reduction of inflammatory mediators by the CISACN treatment in ALI improves its prognosis. Similar results were observed by de França et al. (2021), who demonstrated an anti-inflammatory effect of an MBHA (ISACN, 2-(3-hydroxy-2-oxoindolin-3-yl) chemically like CISACN that negatively modulated the production of IL-1β, TNF-α, and IL-6 by macrophages. In addition, the other studies have demonstrated that other MBHAs present antitumor effects by reducing the production of this group of cytokines (Faheina-Martins et al. 2017; de França 2021; da Silva et al. 2023).

In ALI/ARDS, diffuse alveolar damage is a common histological pattern in patients and occurs due to injury to the epithelial cells, resulting in pulmonary edema, inflammation, hemorrhage, hyaline membrane deposition, and alveolar congestion (Gao et al. 2024; Wang et al. 2024a, b). In LPS-induced lung interstitial inflammation, we found that CISACN treatment improved the histopathological changes including edema, cellular infiltrate, and hemorrhage.

Therefore, the mechanisms of action of CISACN in the ALI model were investigated by examining the inflammatory signaling pathways. The molecular docking technique was used and analyzed the potential binding of CISACN to molecular targets, including TLR4, NF-кB, the p38 MAPK and ERK 2 (Zhai et al. 2022; Voloshin et al. 2023; Kadam and Schnitzer 2024).

The lipopolysaccharide stimulus used to induce ALI binds to the TLR4/MD2 complex, triggering a series of intracellular events that activate MAPKs and NF-кB, which culminate in the production of a range of inflammatory mediators (Okan et al. 2023). Thus, in molecular docking analyses, we observed that CISACN presented high binding energy with TLR4 and p38 MAPK, better than that observed with dexamethasone. Therefore, we proceeded with the study focusing on p38-MAPK. Using a macrophage cell line, we showed that CISACN did not trigger inflammatory mediators, or cause cytotoxicity and decreased the p38 MAPK activity. Corroborating with these data, Da Silva et al. (2023) demonstrated that the ISACN, a similar structure of the CISACN, however, without the hydroxyl methyl, reduced the inflammatory mediators by inhibiting the TLR4/p38 signaling pathway (da Silva et al. 2023).

This study has several limitations, it was conducted only in BALB/c mice, at a single time point (6 h post-LPS), with three doses and no full dose–response curve. The TLR4/p38 MAPK mechanism lacks confirmatory studies (knockout, inhibitors, siRNA), and off-target effects were not evaluated. No pharmacokinetic (ADME) data were obtained. Only oral administration was tested. Nonetheless, the consistency of the anti-inflammatory effects across multiple independent parameters, cell migration, cytokine levels, edema, histopathology, and in vitro assays, provides robust internal validation of the findings.

The convergence of in vivo*, *in silico*,* and in vitro data further strengthens confidence in the proposed TLR4/p38 MAPK mechanism and supports the relevance of CISACN as a promising lead compound. Future studies should address these points to advance CISACN’s therapeutic potential.

## Supplementary Information

Below is the link to the electronic supplementary material.

Supplementary Material 1

## Data Availability

No datasets were generated or analysed during the current study.
