# Supplementary material for: Anti-inflammatory potential of the CISACN adduct in LPS-induced murine models
Source: Inflammopharmacology. 2026 Jun 2;34(7):4867–77. doi: 10.1007/s10787-026-02280-9 (PMC13391798; doi:10.1007/s10787-026-02280-9)
Supplement: Supplementary file 1 — Supplementary Material 1 [file 10787_2026_2280_MOESM1_ESM.docx]

**Anti-inflammatory potential of the CISACN adduct in LPS-induced murine models**

**Inflammopharmacology - Experimental and Therapeutic Studies**

Louise M. de Lima^a^, Francisco A. A. F. Gadelha^*d^, Laércia K. D. Paiva Ferreira^e^, Cosmo I. D. Vieira^a^, João B. de Oliveira^a^, Larissa A. M. Paiva Ferreira^a^, Matheus Victor de Souza Laurentino^a^, Bruno Vinícius da Silva Moura^a^, Teresa Carolliny M. L. Rodrigues^b^, Tayná R. Olegário^c^, Luciana Scotti^b^, Marcus T. Sccotti^b^, Claudio G. Lima-Junior^c^, Naiara N. Dejani^a^, Marcia R. Piuvezam^a^

^a^*Laboratório de Imunofarmacologia, Programa de Pós-graduação em Produtos Naturais e Sintéticos Bioativos, Centro de Ciências da Saúde, Universidade Federal da Paraíba, João Pessoa, PB, Brazil*

*^b^Laboratório de Quimioinformática, Programa de Pós-graduação em Produtos Naturais e Sintéticos Bioativos, Centro de Ciências da Saúde, Universidade Federal da Paraíba, João Pessoa, PB, Brazil*

*^c^Laboratório de Síntese Orgânica Medicinal da Paraíba, Departamento de Química, Centro de Ciências Exatas e da Natureza, Universidade Federal da Paraíba, João Pessoa, PB, Brazil*

*^d^* ^a^*Laboratório de Imunofarmacologia, Instituto de Pesquisa em Fármacos e Medicamentos, Universidade Federal da Paraíba, João Pessoa, PB, Brazil*

*^e^ Complexo de Ciências Médicas e de Enfermagem, Universidade Federal de Alagoas, Arapiraca, AL, Brazil.*

* Author for correspondence: Francisco Allysson A. F. Gadelha, Laboratory of Immunopharmacology, 55+ 83 32167246, João Pessoa, PB, Brazil, Zip Code 58051-900, email: allyssongadelha@ltf.ufpb.br

*3.3 Effect of CISACN on intracellular target molecules*

The binding energy of CISACN and dexamethasone with the molecular targets are expressed in Table 1. CISACN exhibited significant binding energy with p38 MAPK: -72.990 kcal/mol with slightly higher binding energy compared with dexamethasone, 53.035 kcal/mol (Table 1).

The CISACN formed several alkyl interactions with amino acid residues LEU104, ILE84, LYS53, LEU167, ALA51, and VAL38 and a Pi-Cation interaction with LYS53, and van der Waals interactions with VAL105, VAL52, LEU86, LEU75, THR106, HIS107, MET109, ASP168, CYS39, while dexamethasone interacted, by hydrogen and a halogen binding with ASP168, by van der Waals interactions with ARG173, HIS107, GLU71, THR106, MET109, ILE84, LEU75, LEU86, LEU104, VAL105, VAL52, by alkyl interactions with LYS53 and ALA51, and by pi-alkyl interactions with the aromatic ring of residue TYR35 (Fig.5).

The binding energy of CISACN with ERK2 MAPK (Table 1) was -58.329 kcal/mol, lower than the binding energy of dexamethasone (-81.764 kcal/mol). CISACN interacted to ERK2 through hydrogen binding with MET106 and ASP104, carbon-hydrogen with VAL37, alkyl interactions with LEU154, CYS164 and ALA50, and van der Waals with ASP165, LYS52, VAL102, VAL37, ILE82, ILE51, ILE29 and GLN103. In turn, dexamethasone interacted by hydrogen binding with MET106, ASP104, ALA50 and ILE101, alkyl interactions with LEU154, CYS164 VAL37, ALA50 and ILE82, and van der Waals interactions with ILE29, ASP109, LYS112, VAL102, GLU107, THR108 LEU155 and LEU105 (Fig. 5).

The binding energy of CISACN with the p65-NF-кB was -69.387 kcal/mol. In contrast, dexamethasone was -85.147 kcal/mol (Tab.1). CISACN interacted with 65 portions of NF-кB through hydrogen attaching to ARG295, carbon-hydrogen, and pi-cation binding to ASP294, alkyl binding to LEU133, ILE103, VAL137, and ARG136, and van der Waals binding to ASP293 AND HIS296. While the molecule had only one hydrogen bond, dexamethasone had six bonds: two with residue ASP294 and SER205, one with THR292 and GLY208, also a carbon-hydrogen bond with VAL137, as well as alkyl interactions with amino acid residues VAL137 and ARG136 and van der Waals with LEU133, ILE103, ARG295, LEU207, ASP291, ASP293, GLY209 and HIS217 (Fig. 5).

In addition, CISACN interacted with the TLR4 receptor with a score energy of -22.512 kcal/mol, due to the presence of a positive score, and dexamethasone despite being -9.478 kcal/mol, showed little significance because in the Rerank score, it showed a positive value (20.542 kcal/mol) (Table 1). CISACN interacted with TLR4 through conventional hydrogen binding ARG90, and van der Waals interactions with SER120, PHE121, PHE119, and GLU92, whereas dexamethasone interacted with several residues, ARG90 by hydrogen interaction; PHE121 by pi-alkyl interaction; ILE52 by alkyl interaction; SER120 by halogen interaction, and van der Walls interactions with LYS122, LEU61, and GLU92. In addition, dexamethasone showed two unfavorable interactions with residue PHE119 (Fig. 5).

**
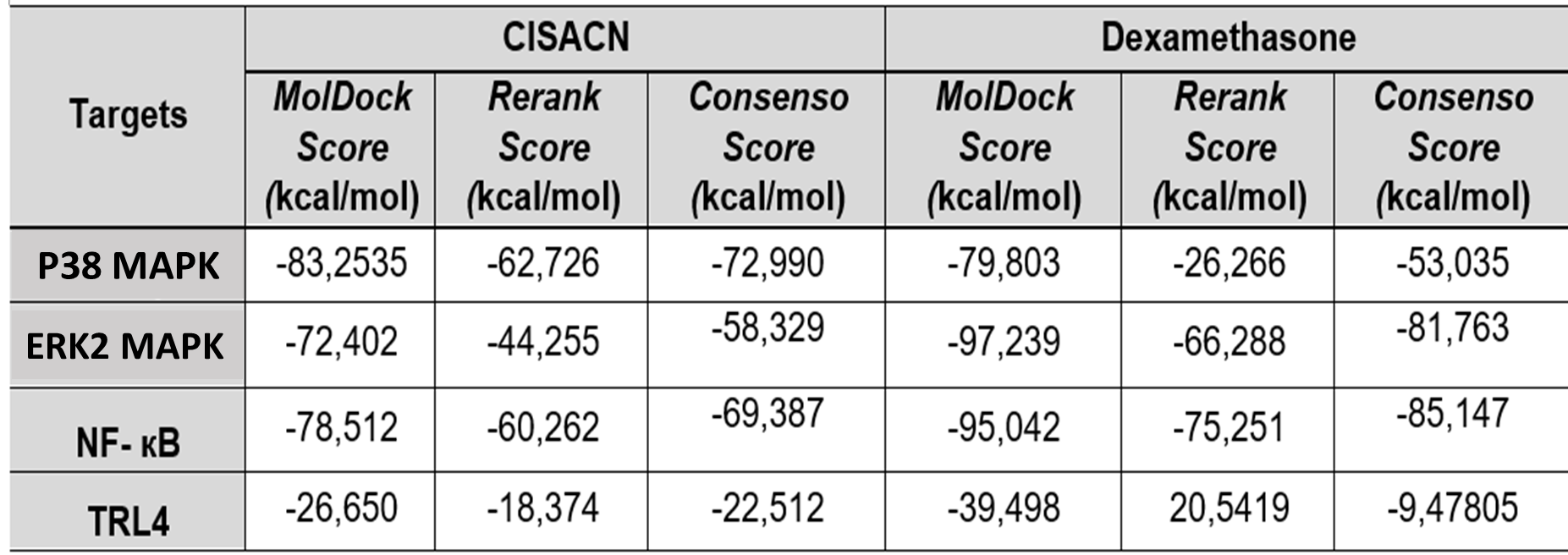
Table 1.** Binding energy of CISACN and dexamethasone on intracellular targets analyzed by MolDock score and Rerank score.

**Figure 5**

**A)**


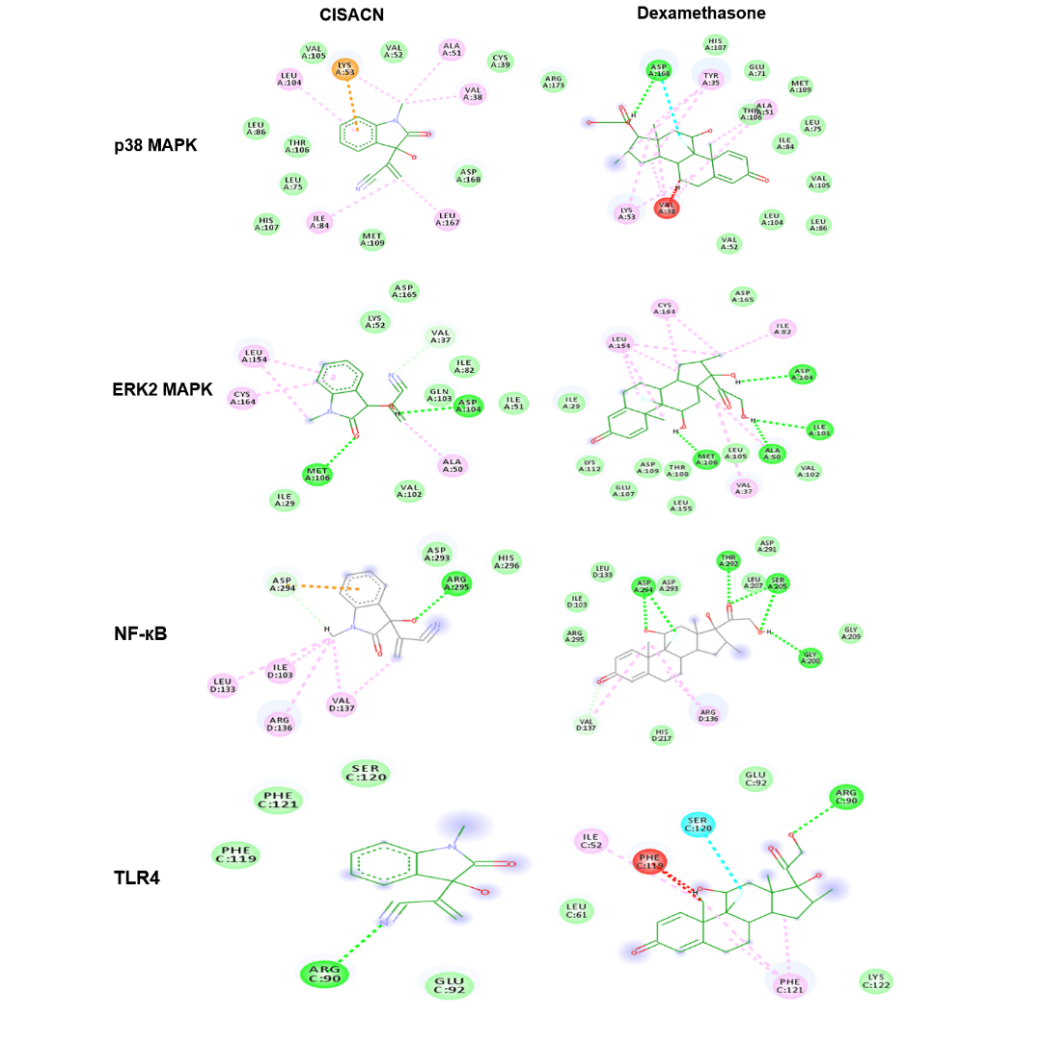


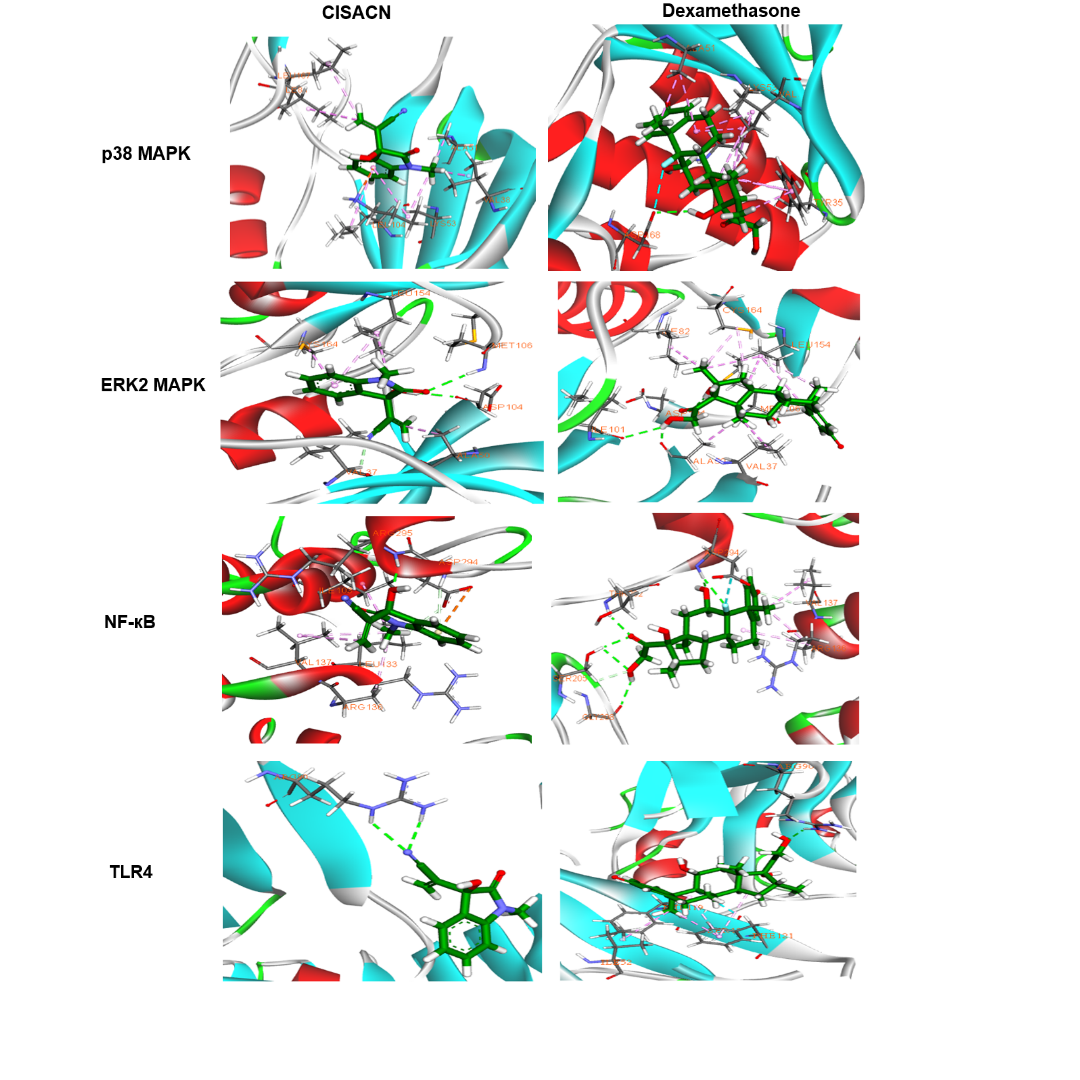
**B)**

**Figure 5.** Molecular docking analysis of CISACN with molecular targets related to the acute lung injury signaling pathway. Interactions of CISACN and dexamethasone in 2D models **(A)** and 3D models **(B)** with the p38 MAPK, ERK2, NF-кB and TLR-4. The software Marvin Sketch 21.13, HyperChem 8.0.6 and Molegro Virtual Docker were used to structure definition and docking analysis.
